# Placenta Praevia with Abnormal Adhesion—A Retrospective Study

**DOI:** 10.3390/clinpract15020023

**Published:** 2025-01-23

**Authors:** Lucian Șerbănescu, Dragoș Brezeanu, Cătălin Nicolae Grasa, Sebastian Mirea, Paris Ionescu, Vadym Rotar, Traian-Virgiliu Surdu, Andreea Cristina Costea

**Affiliations:** 1 Faculty of Medicine, Ovidius University of Constanta, 900470 Constanta, Romania; brezeanudragos@gmail.com (D.B.); catalingrasa@yahoo.com (C.N.G.); paris.ionescu@yahoo.com (P.I.); rotarvadym.obst.gyn@gmail.com (V.R.); traian@surdu.ro (T.-V.S.); acostea100@yahoo.com (A.C.C.); 2 County Clinical Emergency Hospital “Sf. Ap. Andrei”, 900591 Constanta, Romania; sebastian.mirea@umft.ro; 3 Department of Rehabilitation, Balneal and Rehabilitation Sanatorium of Techirghiol, 906100 Techirghiol, Romania

**Keywords:** placenta accreta, placenta praevia, caesarean section, hysterectomy

## Abstract

**Background:** Placenta accreta spectrum (PAS) refers to abnormal placental attachment, categorized into placenta accreta, increta, and percreta, with varying severity. The incidence of PAS has risen alongside the increasing rate of caesarean sections. PAS is a significant cause of maternal complications, including bleeding, hysterectomies of necessity and intestinal or urinary surgical complications, and of foetal complications, preterm birth or foetal anaemia. Early diagnosis is crucial for its management and for improving its outcomes. **Materials and Methods:** This retrospective study, conducted at the County Emergency Clinical Hospital “Saint Andrew the Apostle”, Constanța, analysed cases of placenta praevia and PAS from 2018 to 2022. Data were collected from observation sheets and operative protocols, involving 13,841 patients. Placenta praevia and PAS were diagnosed using ultrasound and MRI and confirmed by histopathology. **Results:** Among the 13,841 deliveries, 25 cases of placenta praevia (0.82% incidence) and 17 cases of PAS (0.57% incidence) were identified. Ultrasound demonstrated 88% sensitivity, and MRI 94% sensitivity for PAS detection. Of the 17 PAS cases, 11 were diagnosed as placenta accreta, 3 were diagnosed as placenta increta, and 3 as placenta percreta, with all percreta cases involving bladder invasion. Hysterectomy was the standard surgical treatment. **Discussion:** The risk factors for PAS included previous caesarean sections (94.1% of PAS cases), smoking, and uterine fibroids. The study confirmed the importance of early imaging and the involvement of a multidisciplinary team in managing PAS, particularly in complex cases with bladder involvement. Caesarean section followed by hysterectomy was the preferred surgical approach. **Conclusions:** Smoking, uterine scars, and uterine fibroids are significant risk factors for placenta praevia with pathological adhesion. Ultrasound and MRI are highly accurate in diagnosing PAS, with histopathology providing definitive confirmation. Multidisciplinary care is essential in managing complex cases, ensuring optimal maternal and foetal outcomes. The surgical treatment involves caesarean section and hysterectomy, with additional interventions for bladder invasion in percreta cases.

## 1. Background

The word accreta comes from the Latin language, where accrescere means to adhere, to attach, this pathology being described for the first time in specialized literature in 1930 [1,2].

Depending on the thickness of trophoblastic invasion, the placenta accreta spectrum (PAS) is divided into placenta accreta, placenta increta and placenta percreta. The most severe, but also the least common form of placental invasion is placenta percreta, with a ratio of the three forms of 80:15:5 [3,4].

Because the terms accreta, increta and percreta, used in the past, do not provide a correct picture of the level of implantation from the histopathological point of view, the International Federation of Obstetrics and Gynaecology (FIGO) proposed another division of PAS into three degrees of severity, the third degree being in turn divided into another three subcategories (A—deep invasion > 75% of the uterine wall; 3D—disruption of the uterine serosa; 3E—extrauterine invasion) [5].

The main risk factors for this type of abnormal placental invasion are placenta praevia in the personal pathological history and previous caesarean delivery. The patients with placenta praevia have a risk of developing an adhesion defect of around 3.3–4%, while in patients with placenta praevia and a previous caesarean section, the risk reaches 50–67% [6].

An increased risk of developing a placenta accreta spectrum pathology was also observed in smoking patients, those with a short interval between two caesarean births or those in whom assisted reproduction techniques were used [7,8].

Given the fact that, in recent years, the completion of birth by caesarean section has experienced a worrying increase, there has been an increase in the incidence of the pathology of placenta accreta spectrum [9].

The symptomatology of this kind of pathology, whether we are talking about the placenta accreta spectrum or positional defects of the placenta, such as praevia placenta, primarily includes vaginal bleeding. This occurs with a frequency of approximately 5% in the second trimester of pregnancy and up to 50% in the third trimester [10].

In exceptional situations of severe aberrant implantation of the percreta type, urinary symptoms may appear, such as macroscopic haematuria or dysuria if placental invasion occurred anteriorly, or rectorrhagia or melena symptoms in the case of patients in whom placental invasion occurred posteriorly, involving the small intestine or the large intestine [11].

In case of vaginal bleeding, a differential diagnosis of PAS or placenta praevia must be made in the following situations: abruptio placentae, vasa praevia, uterine rupture, cervical pathology and traumatic injuries of the cervix, premature rupture of membranes, premature labour or traumatic lesions of the vaginal wall.

The clinical diagnosis of placenta praevia cannot be established with certainty without a pathological examination. The presence of PAS can be clinically suspected if the patient presents to the emergency unit complaining of spontaneous vaginal bleeding that occurred after 24 weeks of amenorrhea, unaccompanied by painful uterine contractions, and, when examining the patient, the basal uterine tone is normal [10].

Morphological screening in the second trimester performed between 20 and 22 weeks can establish the location, grade, placental echogenicity, as well as the normal or pathological insertion of the umbilical cord [12].

The diagnosis of this type of pathology can be made either with ultrasound or by MRI. According to the specialized literature, the diagnosis can be made with the help of obstetrical ultrasound, some studies even conferring a higher or equal sensitivity to the ultrasound analysis compared to the MRI exam. The sensitivity and specificity in the case of the ultrasound examination can go up to 92 and 86%, respectively, whereas those of the MRI examination can reach 93% and 91%, respectively [13,14]. The final diagnosis in terms of adhesiveness is made by a histopathological examination that can establish with microscopic accuracy the degree of penetrability and trophoblastic invasion [14,15].

Placenta percreta complications involving the mother that can be mentioned include the necessity of hysterectomy, appreciable bleeding and disseminated intravascular coagulopathy or multiple organ failure, which can ultimately lead to maternal death [16].

Foetal complications include preterm birth, intrauterine growth restriction and intrauterine foetal death.

A case where placenta accreta spectrum is suspected will be admitted to a tertiary centre with the permanent presence of a gynaecologist, an anaesthetist and a surgeon.

In the situation of a surgical intervention in a patient suspected of having placenta percreta with bladder or ureteral invasion, the presence of a urologist is also necessary.

The American College of Obstetricians and Gynaecologists recommends, in an effort to increase patient safety, that the surgical intervention be performed by a team of experienced obstetricians and also that surgeons from other specialties such as urology or general surgery be included [17,18].

If the presence of a suspected placenta accreta spectrum pathology, it is preferable that the delivery be performed between 34 and 36 weeks of pregnancy, with completion of the delivery by elective caesarean section, followed by hysterectomy if necessary [19].

Before the surgical intervention, it is necessary to ensure that the intensive care unit is capable to handle this type of cases, 3–4 units of packed red blood cells and fresh frozen plasma are stored and prepared for immediate transfusion and also some fast-acting haemostatic drugs, such as recombinant factor VII—NoVo Seven—are available [19].

Some authors consider that a conservative treatment of placental adhesion pathologies might be useful, although this option can be burdened by major complications such as appreciable haemorrhage, endometritis and recurrence of placenta accreta spectrum pathology [14].

## 2. Materials and Methods

The present study aimed to analyse the incidence, the etiological factors, the diagnostic and therapeutic methods and the social implications of placenta praevia and placenta accreta spectrum in the County Emergency Clinical Hospital “Saint Andrew the Apostle”, Constanta, within the Obstetrics-Gynaecology II department. The study was approved by the Ethical Committee of II Obstetrics-Gynaecology Clinic of the “Sf Apostol Andrei”, Constanta County Emergency Clinical Hospital (approval no. 004/01.12.2023) and complied with the revised ethical guidelines of the Declaration of Helsinki. Informed consent was obtained from all subjects involved in the study. All patients admitted to our clinic gave their consent for their clinical data to be included in studies respecting their confidentiality.

The study was retrospective, covering a 5-year period between 1 January 2018 and 31 December 2022. The data were collected from the department’s observation sheets and operative protocols. Between 1 January 2018 and 31 December 2022, 13,841 patients were admitted to the Obstetrics and Gynaecology department.

A breakdown was made of the main etiological factors involved, the degree of cotyledon penetration, the surgical therapy practiced and the severity of the clinical forms of the placenta accreta cases. Particular attention was paid to the cases of placenta percreta and the complications generated in these cases.

### Data Analysis

The statistical analysis was performed using IBM SPSS version 23. The data are presented as mean ± standard deviation (SD) for continuous variables in cases of symmetric distributions. The normality of the continuous data was estimated with Shapiro–Wilk tests of normality. For hypotheses testing, independent sample median test, *t*-Student test and chi-square test of association were used depending on the type of the analysed variables. The significance level α was set at 0.01. If the test statistics for every test conducted was in the critical region and the *p*-value was less than or equal to the significance level, we decided to reject the null hypothesis in favour of the alternative hypothesis. In the Discussions section, we present the *p*-statistic and the confidence interval only when they had an important statistical significance in terms of our observations and future conclusions. Although the number of cases studied was not very large, the accuracy of the study is significant.

## 3. Results

Among the 6708 patients hospitalized for childbirth assistance, 25 presented the diagnosis of placenta praevia complicated with bleeding. The total incidence was 0.82% (Table 1).

Among the 6708 patients hospitalized for childbirth assistance, 17 presented the diagnosis of placenta accreta spectrum. The total incidence was 0.57% (Table 1).

Of the 17 cases of PAS, 11 cases were of placenta accreta, 3 of placenta increta and 3 of placenta percreta (Table 2).

The sensitivity of the MRI was 94.1%, and that of the echocardiogram was 88% (Table 3).

## 4. Discussion

The sensitivity and specificity of ultrasound examinations can reach up to 92% and 86%, respectively, compared to those of MRI examinations, which can reach up to 93% and 91%, respectively, in the detection of cases with abnormal placental penetration [13]. In our study, the sensitivity of the ultrasound examination was 88%, and the specificity was 88%, while for the MRI examination, sensitivity was 94% and specificity was 88%. A higher sensitivity of MRI versus ultrasound examinations and equal specificity were observed (Table 3). Colour and 3D Doppler examinations are important levers of ultrasound examination. It is possible that, in the future, we will encounter similar specificities and sensitivities of the two methods. The final diagnosis in terms of adhesiveness is made by histopathological examination, which can establish the degree of penetrability and trophoblastic invasion with microscopic accuracy [14]. In our study including 17 cases, anatomopathological examination revealed a placenta accreta–increta–percreta pathology. When placenta accreta spectrum pathology is suspected, the patient will be admitted to a tertiary centre with the permanent presence of a gynaecologist, an anaesthesiologist, and a surgeon. If bladder or ureteral invasion is suspected, the presence of a urologist is also necessary [19]. All of the percreta cases in our study that involved the urinary bladder were confirmed by cystoscopy, and the presence of the urologist in the multidisciplinary team was necessary. Total hysterectomy and suture of the bladder were enough to solve all the cases. An important challenge is posed by the postoperative recovery of these patients. Sometimes, especially in cases of placenta increta with bladder penetration, the surgical team may have to sacrifice an important area of the bladder or compromise its innervation, which can predispose to involuntary postoperative urine loss. In these cases, it is more than necessary to postoperatively recover the patients and subsequently re-educate the sphincter by practicing Kegel exercises. Our experience with such forms of recovery is a gratifying one, two of our previous published studies highlighting the importance of practicing Kegel exercises in patients with involuntary urine loss or with various forms of atrophic colpitis [20,21]. All patients had severe anaemia, with haemoglobin levels below 7 g/dL, and required blood transfusions.

There are also cases in which heavy bleeding can lead to varying degrees of kidney damage. Thus, the collaboration of gynecologist, nephrologist, recovery doctor is more than beneficial and necessary. These cases with severe hemorrhagic anemia often require a postoperative recovery similar to the recovery techniques used in patients with heart disease [22].

The total incidence of placenta praevia in the study group was 0.82%. These data are in line with those in worldwide specialized literature. For example, a 10-year retrospective study at the Cleveland Maternity Hospital shows an incidence of placenta praevia of 0.8% [23]. Similarly, a study conducted in Canada showed an incidence of 0.73% of placenta praevia [24]. Although these studies show a small percentage variation from one another, it is necessary to also consider that some meta-analyses indicate a much lower incidence of placenta praevia, i.e., around 0.15% of the total number of births [25]. As can be seen, there are also studies, not many, that report much lower incidences of placenta praevia.

In our study, it is imperative to note that 2982 patients underwent caesarean section, which corresponded to 44.5% of the total births. Of the 25 cases of placenta praevia, 18 were patients with a history of uterine scarring, accounting for 72%. Among the 25 placenta praevia cases, 17 were diagnosed as placenta accreta, representing 68% of the total cases. Within the 17 PAS cases, 11 involved invasion-type placenta accreta, 3 involved invasion-type placenta increta, and another 3 involved invasion-type placenta percreta, amounting to 17.6%. In all of this cases of placenta accreta (64.7%), increta (17.6%) and percreta (17.6%), total hysterectomy was performed after the caesarean section. The distribution by gestational and parity grades did not yield significant data. However, it is crucial to consider additional etiological factors such as smoking, age, and alcohol consumption. In our study on placenta accreta spectrum, the overall percentage of smoking patients was 82.35%, i.e., 72.72% for the placenta accreta cases and 100% for the placenta increta and percreta cases (Table 2). In addition, 54.54% of the patients with PAS spectrum consumed alcohol in moderate amounts (21–40 g of pure alcohol/day). In relation to the aetiopathogenic mechanism associated with the development of the placenta praevia pathology, a set of factors can be identified as having presented suggestive evidence and are considered major predisposing factors for the emergence of this anomaly. These factors include the history of caesarean section deliveries, an advanced maternal age, smoking, abortions, and pregnancy with male foetuses. Additionally, it is acknowledged that pregnancy-induced hypertension and preeclampsia are not contributing factors [26].

The percentage of patients over 35 years of age was 40% in the group of patients with placenta praevia and 41% in the PAS group. In women over the age of 35, there has been a noticeable increase in the incidence of placenta praevia, a condition that can lead to severe complications during delivery, including life-threatening haemorrhages. This increase may be attributed to various factors such as changes in the uterine structure, like the presence of fibroids, endometrial alterations, chronic endometritis and other related conditions. Age-related structural changes in the uterine lining may disrupt the normal placental implantation. Additionally, older women often have a history of multiple pregnancies, miscarriages, or uterine surgery, all of which can increase the risk of placenta praevia.

Our study found that 94.1% (*p* < 0.01, CI 95%) of the patients in the abnormal placental adhesion group had previous caesarean sections, a value significantly higher than that of 44.5% of the general incidence and of 72% (*p* < 0.01, CI 95%*)* of the caesarean section incidence in the placenta praevia group. A previous second caesarean section was found in 36.3% of the cases of PAS. Uterine scarring from prior surgeries appears to influence placental positioning, as scarring near the cervix may lead to the implantation of the placenta in lower segments, increasing the risk of placenta praevia. With each additional caesarean delivery, the likelihood of placenta praevia grows due to the accumulating scar tissue, particularly in women with multiple prior caesareans [27].

A potentially helpful intervention in managing these cases is uterine artery embolization (UAE), which has been found to reduce the haemorrhage risk and limit the need for blood transfusions. UAE is a valuable alternative to traditional caesarean hysterectomy, particularly when uterine preservation is desired. Studies have shown that UAE, as part of a standardised protocol, can lower blood loss significantly, thereby supporting better maternal outcomes [28]. In none of the 17 cases of PAS, uterine artery embolization was performed.

In the praevia placenta group, 60% of the children born were male, while in the PAS group, 58.8% of them were male (*p* < 0.01, CI 95%*)*. In the general population, the ratio of boys to girls among newborns is 51%, in Romania. Another observation is the association between pregnancy with a male foetus and placenta praevia, although the reasons are not fully understood. Hypotheses include the influence of the male foetal hormonal environments on placental attachment and possible growth patterns of male foetuses that could contribute to abnormal placental positioning [29]. Furthermore, lifestyle factors like smoking—present in 70.5% (*p* < 0.01, CI 95%*)* of our cases compared to a general Romanian smoking rate of about 55%—can impact placental positioning. Exposure to nicotine and carbon monoxide may lead to chronic placental hypoxia, increasing the risk of placenta praevia [30,31,32].

Although uterine fibroids are not traditionally considered a risk factor, our study found that 30% of the placenta praevia cases were associated with fibroids, which were only observed in women over 30. In the group of patients with PAS, the incidence of fibromatous uterus was also 29.4% (Table 2). Occasionally, pregnant women with placenta praevia report moderate abdominal pain unrelated to contractions, potentially due to vascular compression, including in formations like Buhler’s arc [33]. Abdominal pain can be severe when the neighbouring organs are penetrated; sometimes, this pain can be confused with pain from twisting an ovarian tumour formation or from the bursting of an ovarian cyst [34]. Moreover, learning about the presence of placenta praevia during the second trimester morphology scan can cause considerable stress, similar to the distress experienced by traumatized patients [35].

In our study, all placenta praevia cases were successfully managed by caesarean section without rupture of the placental blood vessels. Maternal anaemia was mild to moderate overall, except in cases of placenta percreta.

The anatomopathological examination of the placentas was essential in establishing the diagnosis and in classifying the cases as placenta accreta, increta or percreta. Additional examinations performed on these placentas can also include the analysis of the placental vascularization; injection of plastids and subsequent corrosion of the preparations can provide important information about placental vascularization in these cases [36].

## 5. Conclusions

Smoking, uterine scars and uterine fibroids remain the most important etiological factors involved in placenta praevia with pathological adhesion.

The incidence of placenta praevia and PAS is higher in women pregnant with male foetuses.

The accuracy of ultrasound and MRI imaging was relatively good, with only a few cases requiring a multidisciplinary team for the management of percreta placentas affecting both the bladder and the rectum, and other investigations such as cystoscopy and recto–colonoscopy. New ultrasound machines and MRI machines offer increasing possibilities for the detection and analysis of these cases.

Caesarean section followed by hysterectomy represented the surgical therapy in the cases of placenta accreta or increta, with additional bladder suture in the cases of placenta percreta with bladder invasion.

A multidisciplinary medical team (gynaecologist, urologist, nephrologist, surgeon and anaesthesiologist) represents a gold standard for managing cases of placenta praevia with pathological adhesion.

## Figures and Tables

**Table 1:** Distribution of patients with placenta praevia and placenta accreta spectrum.

	Births	Caesarean	Placenta Praevia	Placenta Accreta
2018	1214	585	5 (0.85%)	4 (0.68%)
2019	1135	526	4 (0.76%)	3 (0.57%)
2020	1503	576	6 (1.04%)	3 (0.52%)
2021	1477	654	5 (0.76%)	4 (0.61%)
2022	1379	641	5 (0.78%)	3 (0.46%)
	6708	2982	25 (0.82%)	17 (0.57%)

**Table 2:** General distribution of placenta accreta spectrum cases.

	Placenta Accreta 11 Cases	Placenta Increta 3 Cases	Placenta Percreta 3 Cases
Smoking more than 10 cigarettes	8	3	3
Moderate alcohol consumption (21–40 g of pure alcohol/day)	4	1	1
Previous 1 C-section	6	3	3
Previous 2 C-sections	2	1	1
Rectal penetration of cotyledons	-	-	-
Bladder penetration of cotyledons	-	-	3
Penetration of cotyledons in other organs	-	-	-
Hysterectomy of necessity	11	3	3
Uterine fibroma	3	1	1

**Table 3:** Ultrasound and MRI sensitivity and specificity in the diagnosis of placenta accreta spectrum.

	Ultrasound	MRI
Sensitivity	88%	94.1%
Specificity	88%	88%

## Data Availability

The data supporting this study’s findings are available on request from the corresponding author, Lucian Șerbănescu.
